# 

**Published:** 1892-01-09

**Authors:** 


PRICE ONE PENNY.

No. 276, Vol. XI.—January 9th, 1892.

[Registered at the General Post Office as a Newspaper for transmission in the United Kingdom and Abroad.]

WITH EXTRA NURSING SUPPLEMENT.

Per Annum, 6s. 6d., post free.

Monthly Parts, 6d.; post free, 8d.

Ditto per Annum, 8s., post free.

### THE HOSPITAL

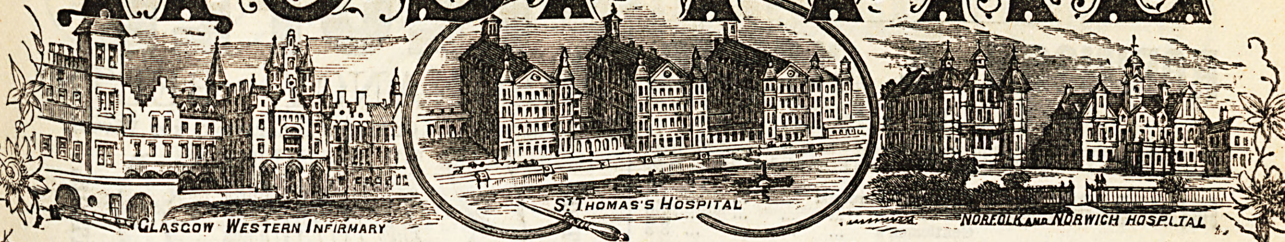

A WEEKLY JOURNAL OF

### Science, Medicine, Nursing, and Philanthropy.

SATURDAY, JANUARY 9th, 1892.

The Doctor's Christmas Box - - - - - - - 177
Passing Topics.—"Obscurantism" - - - - - - - 178
The Doctor's Armchair.—Social Detectives - - - - 179
Some Scientific Aspects of Insanity. VIII.—The Care and Treatment of Melancholia - - - - - - - 180
Some American Hospitals. II.—San Francisco - - - 181
Notes and News - - - - - - - - - 182
General Charities - - - - - - - - 183
Scraps and Gleanings - - - - - - - 183
Annotations.—A Call for Crusaders—A Peerless Peer - - 184
Hospital Obscurantism - - - - - - - - 188
The Practitioner's Mirror and Retrospect—
Medicine.—Effects of Anæmia on the Eye—Diseases of the Arteries, Capillaries, and Veins - - - - - - 185
Surgery.—Acute Mastitis - - - - - - - - 186
Aural Surgery—The Surgical Treatment of Septic Thrombosis and other Complications of the Middle Ear Disease.—II. - - - - - - - - - - 187
New Drugs, Appliances, and Things Medical.—The Claxton Combined Bed, Table, and Book-Rack—Bovril and its Preparations - - - - - - - - - 188
The Student of Medicine—Appointments and Vacancies.

EXTRA SUPPLEMENT.—THE NURSING MIRROR.

En Passant.—St. Andrew's Ambulance Association—Short Items—The Royal National Pension Fund for Nurses — Nursing Notes — Christmas Parcels — The Charge Against the Training Schools - - - - lxxxv

Lectures on Surgical Ward Work and Nursing. Lecture XLI.—Instruments used in Urethrotomy - - lxxxvi

For Reading to the Sick. One Amongst Us - - lxxxvii

Our Indictment—Appointments - - - - - - lxxxviii

Keeping Christmas - - - - - - - - lxxxix

Everybody's Opinion.—Notice of Appointments - - xc

Where to Go - - - - - - - - - xc

Presentations - - - - - - - - - xc

Death in Our Ranks - - - - - - - - xc

Amusements and Relaxation - - - - - - - xc

